# Predicting Long-term Disease-free Survival After Resection of Pancreatic Ductal Adenocarcinoma: A Nationwide Cohort Study

**DOI:** 10.1097/SLA.0000000000006004

**Published:** 2023-07-17

**Authors:** Iris W.J.M. van Goor, Thijs J. Schouten, Daphne N. Verburg, Marc G. Besselink, Bert A. Bonsing, Koop Bosscha, Lodewijk A.A. Brosens, Olivier R. Busch, Geert A. Cirkel, Ronald M. van Dam, Sebastiaan Festen, Bas Groot Koerkamp, Erwin van der Harst, Ignace H.J.T. de Hingh, Martijn P.W. Intven, Geert Kazemier, Maartje Los, Gert J. Meijer, Vincent E. de Meijer, Vincent B. Nieuwenhuijs, Daphne Roos, Jennifer M.J. Schreinemakers, Martijn W.J. Stommel, Robert C. Verdonk, Hjalmar C. van Santvoort, Lois A. Daamen, I. Quintus Molenaar

**Affiliations:** * Department of Surgery, Regional Academic Cancer Center Utrecht, Utrecht University, University Medical Center Utrecht Cancer Center & St. Antonius Hospital Nieuwegein, Utrecht, the Netherlands; † Department of Radiation Oncology, Regional Academic Cancer Center Utrecht, Utrecht University, University Medical Center Utrecht Cancer Center, Utrecht, the Netherlands; ‡ Department of Surgery, Amsterdam UMC, location University of Amsterdam, Amsterdam, the Netherlands; § Cancer Center Amsterdam, the Netherlands; ∥ Department of Surgery, Leiden University Medical Center, Leiden, the Netherlands; ¶ Department of Surgery, Jeroen Bosch Hospital, Den Bosch, the Netherlands; # Department of Pathology, Regional Academic Cancer Center Utrecht, Utrecht University, University Medical Center Utrecht Cancer Center & St. Antonius Hospital Nieuwegein, Utrecht, the Netherlands; ** Department of Medical Oncology, Regional Academic Cancer Center Utrecht, Utrecht University, University Medical Center Utrecht Cancer Center & Meander Medical Center Amersfoort, Utrecht, the Netherlands; †† Department of Surgery, Maastricht UMC+, Maastricht, the Netherlands; ‡‡ Department of Surgery, OLVG, Amsterdam, the Netherlands; §§ Department of Surgery, Erasmus MC Cancer Institute, Rotterdam, the Netherlands; ∥∥ Department of Surgery, Maasstad Hospital, Rotterdam, the Netherlands; ¶¶ Department of Surgery, Catharina Hospital, Eindhoven, the Netherlands; ## Department of Surgery, Amsterdam UMC, location Vrije Universiteit, Amsterdam, the Netherlands; *** Department of Medical Oncology, Regional Academic Cancer Center Utrecht, Utrecht University, University Medical Center Utrecht Cancer Center & St. Antonius Hospital Nieuwegein, Utrecht, the Netherlands; ††† Department of Surgery, University of Groningen, University Medical Center Groningen, Groningen, the Netherlands; ‡‡‡ Department of Surgery, Isala, Zwolle, the Netherlands; §§§ Department of Surgery, Renier de Graaf Gasthuis, Delft, the Netherlands; ∥∥∥ Department of Surgery, Amphia Hospital, Breda, the Netherlands; ¶¶¶ Department of Surgery, Radboud University Medical Center, Nijmegen, the Netherlands; ### Department of Gastroenterology, Regional Academic Cancer Center Utrecht, Utrecht, the Netherlands; **** Imaging Division, University Medical Centre Utrecht, Utrecht University, Utrecht, the Netherlands

**Keywords:** pancreatic ductal adenocarcinoma, recurrence, prognosis, survival, disease-free survival, risk score, model

## Abstract

**Objective:**

To develop a prediction model for long-term (≥5 years) disease-free survival (DFS) after the resection of pancreatic ductal adenocarcinoma (PDAC).

**Background:**

Despite high recurrence rates, ~10% of patients have long-term DFS after PDAC resection. A model to predict long-term DFS may aid individualized prognostication and shared decision-making.

**Methods:**

This nationwide cohort study included all consecutive patients who underwent PDAC resection in the Netherlands (2014–2016). The best-performing prognostic model was selected by Cox-proportional hazard analysis and Akaike’s Information Criterion, presented by hazard ratios (HRs) with 95% confidence intervals (CIs). Internal validation was performed, and discrimination and calibration indices were assessed.

**Results:**

In all, 836 patients with a median follow-up of 67 months (interquartile range 51–79) were analyzed. Long-term DFS was seen in 118 patients (14%). Factors predictive of long-term DFS were low preoperative carbohydrate antigen 19-9 (logarithmic; HR 1.21; 95% CI 1.10–1.32), no vascular resection (HR 1.33; 95% CI 1.12–1.58), T1 or T2 tumor stage (HR 1.52; 95% CI 1.14–2.04, and HR 1.17; 95% CI 0.98–1.39, respectively), well/moderate tumor differentiation (HR 1.44; 95% CI 1.22–1.68), absence of perineural and lymphovascular invasion (HR 1.42; 95% CI 1.11–1.81 and HR 1.14; 95% CI 0.96–1.36, respectively), N0 or N1 nodal status (HR 1.92; 95% CI 1.54–2.40, and HR 1.33; 95% CI 1.11–1.60, respectively), R0 resection margin status (HR 1.25; 95% CI 1.07–1.46), no major complications (HR 1.14; 95% CI 0.97–1.35) and adjuvant chemotherapy (HR 1.74; 95% CI 1.47–2.06). Moderate performance (concordance index 0.68) with adequate calibration (slope 0.99) was achieved.

**Conclusions:**

The developed prediction model, readily available at www.pancreascalculator.com, can be used to estimate the probability of long-term DFS after resection of pancreatic ductal adenocarcinoma.

Pancreatic ductal adenocarcinoma (PDAC) is one of the most life-threatening cancers.^1^ Resection combined with chemotherapy is the only potentially curative treatment option.^2–4^ Although advancements have been made in pancreatic cancer management over the past years, the majority of patients who undergo resection will develop disease recurrence within 2 years after surgery.^4–8^ Consequently, this is the main cause of mortality in these patients and results in a poor 5-year survival rate of only 17% after resection.^9^

Despite the high recurrence rates, a small number of ~10% of patients have a long-term disease-free survival (DFS) of at least 5 years.^10,11^ Due to the restricted number of long-term survivors, little is known about their distinguishing clinical characteristics and tumor biology. Identification of factors associated with long-term DFS may contribute to these insights. In addition, an estimation of patients’ probability of long-term survival may aid prognostication and shared decision-making for individual patients after resection of PDAC.

Many factors associated with PDAC recurrence have been identified previously, such as age, preoperative serum carbohydrate antigen 19-9 (CA 19-9) level, differentiation grade, vascular resection, perivascular invasion, resection margin status, lymph node status and ratio, tumor size/stage, and adjuvant chemotherapy.^8,10,12^ However, these studies focused solely on identifying factors associated with disease recurrence in general or with recurrence within 5 years after resection of PDAC. Little is known about prognostic factors associated with a DFS of at least 5 years. The one study that did investigate predictors for 5-year DFS only found an absence of perineural invasion to be correlated.^11^

This study aimed to identify prognostic factors and to develop a prediction model for long-term DFS (ie, ≥ 5 y) after the resection of PDAC.

## METHODS

### Study Design

A nationwide observational study was performed in all Dutch centers performing pancreatic cancer surgery. All consecutive patients who underwent surgical resection between 2014 and 2016, as registered within the mandatory Dutch Pancreatic Cancer Audit (DPCA), were included.^13^ Patients were excluded in case of complication-related mortality within 90 postoperatively, as this was not related to disease recurrence. The study protocol was discussed and approved during a plenary meeting of the scientific committee of the Dutch Pancreatic Cancer Group, and institutional board approval of each participating center was obtained.^14^ We adhered to the ‘Transparent Reporting of a multivariable prediction model for Individual Prognosis or Diagnosis’ (TRIPOD) and the ‘Strengthening the Reporting of Observational Studies in Epidemiology’ (STROBE) guidelines.^15,16^

### Data Collection and Predictor Selection

Baseline and perioperative data were obtained from the DPCA database. Additional data on postoperative complications, follow-up, disease recurrence, and survival were retrieved from the patients’ hospital records.

On the basis of their previously suggested association with PDAC recurrence by literature, candidate predictors selected for model development were sex (male or female), Charlson Age-adjusted Comorbidity Index (CACI) at diagnosis (< 4 or ≥ 4), neoadjuvant therapy (yes or no), preoperative serum CA 19-9 level (continuous), vascular resection (yes or no), major complications (yes or no), tumor stage (T1, T2 or T3), tumor differentiation (well/moderate or poor), lymphovascular invasion (yes or no), perineural invasion (yes or no), lymph node stage (N0, N1 or N2), resection margin status (R0 or R1), and adjuvant chemotherapy (yes or no). In addition, the prognostic value of the number of cycles of adjuvant chemotherapy received was evaluated.

The MDCalc CACI calculator was used to determine the CACI, and it was dichotomized into < 4 or ≥ 4 based on previous studies.^17–20^ Patients’ preoperative CA 19-9 level was transformed on a logarithmic scale to achieve a normal distribution. Vascular resection could be either venous, arterial, or both. Any complications that required surgical or radiologic intervention or intensive care unit admittance, or either led to single-organ or multi-organ failure or patient mortality, were scored as major complications. The eighth edition of the American Joint Committee on Cancer TNM guidelines was used to determine tumor (T) stage, lymph node (N) status, and TNM status.^21^ Resection margin status was considered microscopically positive (R1) if tumor cells were present within 1 mm of the closest resection margin, apart from the anterior surface.^22^

### Outcomes

The primary outcome was a DFS ≥ 5 years after the resection of PDAC. DFS was defined as the time between the date of surgery and the date of recurrence diagnosis. Patients without disease recurrence were censored at the date of last follow-up. Disease recurrence was diagnosed by histologic confirmation or when this was absent by consensus at a multidisciplinary meeting based on results of imaging and serum tumor marker testing. During the study period, a symptomatic postoperative follow-up was considered standard in the Netherlands.^22^ In case patients encountered symptoms suspicious of disease recurrence, conduction of serum tumor marker testing and imaging was indicated. Only if patients participated in a study with a standardized surveillance strategy or if the patient or treating clinician preferred a standardized surveillance strategy, serum tumor marker testing and imaging were performed in a standardized fashion.

### Statistical Analysis

Baseline characteristics were presented using descriptive statistics. χ^2^ or Fisher exact test is used to compare categorical variables as appropriate. Parametric continuous variables are presented as mean with standard deviation and are compared using the Student *T*-test. Nonparametric continuous variables are presented as median with interquartile range (IQR) and are compared using the Mann-Whitney-*U* test. Multiple imputations with the iterative Markov chain Monte Carlo method (5 imputations; 10 iterations) were used to account for missing baseline data, which were considered missing at random.^23^ Variable inflation factors were calculated to rule out multicollinearity between predictors. Kaplan-Meier survival curves were used to assess DFS and overall survival (OS), which were reported as median with 95% confidence intervals (95% CIs).

Outliers were identified by calculating the difference in beta (Dfbeta) residuals, and proportionality was examined by calculating Schoenfeld residuals. If the proportional hazard assumption did not hold, variables were transformed accordingly. Multivariable Cox-proportional hazard analysis was performed to identify factors that were independently associated with a DFS of at least 5 years. Resulting hazard ratios (HRs) with corresponding 95% CIs and probability values (P) were reported. HRs greater than 1 were associated with DFS of 5 years or more. Akaike’s information criterion was used to select the final prediction model. Internal validation in 1000 bootstrap samples was performed to correct for optimism. Discriminative ability was assessed by the concordance index (*C*-index), whereby an index of 1 defines perfect discrimination. Calibration was assessed by the slope of calibration plots, in which an intercept of 0 combined with a slope of 1 corresponds to perfect calibration.

On the basis of the final model, a risk score was calculated. Each of the included predictors was assigned a certain number of points based on the corresponding HRs, which collectively yield a total score. This total score was directly translated into an individual 5-year DFS probability. A receiver operating characteristic curve was plotted to evaluate the prognostic ability. An online calculator based on the developed model was made available on www.pancreascalculator.com.

All statistical analyses were performed using an *R* language environment (version 4.1.2, naniar, dplyr, tidyr, plotrix, mice, rms, MASS, survminer, pROC, PredictABEL packages; http://R-project.org). A 2-sided *P* of < 0.05 was considered statistically significant.

### Sensitivity Analysis

Since a standardized follow-up was considered standard in the Netherlands, only limited patients will be long-term survivors confirmed by imaging or CA 19-9 testing. After the development of the final model, the discriminative ability of this model was evaluated by determining the *C*-index and calibration slope of this model among the confirmed long-term disease-free survivors. Those were defined as patients who had imaging with absence of disease recurrence or low CA 19-9 values at 5 years after their resection of PDAC or thereafter (confirming their long-term DFS). Patients who were long-term survivors based on the absence of symptoms were excluded from this analysis.

## RESULTS

A total of 836 patients who underwent resection of PDAC were included (Supplemental Table 1, Supplemental Digital Content 1, http://links.lww.com/SLA/E729). Median follow-up was 67 months (IQR 51–79 mo) for patients still alive at the end of the study. In the minority of patients, a standardized surveillance with routine CA 19-9 testing (n = 84, 10%) and/or routine imaging (n = 110, 13%) was applied. Median OS of the entire cohort was 21 months (95% CI 19–24 mo).

**TABLE 1:** Descriptive Statistics Comparing Long-term Disease-free Survivors of Pancreatic Ductal Adenocarcinoma to Remaining Study Population

	DFS ≥ 5 y (n=118)	DFS < 5 y (n=718)	*P*
Male sex, n (%)	67 (57)	392 (55)	0.66
CACI, n (%)	—	—	0.27
<4	66 (56)	362 (50)	—
≥4	52 (44)	356 (50)	—
Neoadjuvant therapy, n (%)	15 (13)	57 (8)	0.09
Preoperative serum CA 19-9 level, median (IQR), U/mL	52 (18–186)	127 (33–485)	<0.001
Vascular resection, n (%)	22 (19)	203 (28)	0.03
Tumor stage 8th AJCC edition, n (%)	—	—	<0.001
T1	27 (23)	64 (9)	—
T2	66 (57)	449 (64)	—
T3	23 (20)	190 (27)	—
Tumor differentiation, n (%)	—	—	0.01
Well/moderate	82 (80)	429 (67)	—
Poor	21 (20)	212 (33)	—
Perineural invasion, n (%)	77 (79)	575 (90)	0.001
Lymphovascular invasion, n (%)	42 (49)	387 (69)	<0.001
Lymph node status 8th AJCC edition, n (%)	—	—	<0.001
N0	64 (55)	179 (25)	—
N1	44 (38)	282 (39)	—
N2	9 (8)	255 (36)	—
Resection margin status, n (%)	—	—	<0.001
R0 ≥1 mm	74 (64)	323 (45)	—
R1 < 1 mm	42 (36)	388 (55)	—
Major complications, n (%)	28 (24)	222 (31)	0.11
Adjuvant chemotherapy, n (%)	85 (75)	446 (64)	0.03

AJCC indicates American Joint Committee on Cancer; CA 19-9, carbohydrate antigen 19-9; CACI, Charlson Age-adjusted Comorbidity Index; DFS, disease-free survival; P, probability-value.

## Disease Recurrence

In all, 613 patients (73%) developed disease recurrence with a median DFS of 16 months (95% CI 15–19 mo). Of these, 295 patients (48%) developed early recurrence within 12 months after surgery. At 5 years after surgery, 118 patients (14%) were disease-free.

Comparison of the long-term survivors to the remaining study population revealed lower median preoperative serum CA 19-9 level (52 vs. 127 U/mL; *P* < 0.001) and less often performed vascular resections (19% vs. 28%; *P* = 0.03) in these patients. Also, the average tumor size was smaller (T1 23% vs. 9%, T2 57% vs. 64%, and T3 20% vs. 27%; *P* < 0.001), and tumors were more often well/moderately differentiated (80% vs. 67%; *P* = 0.01). Perineural and lymphovascular invasion was observed less often (79% vs. 90%; *P* = 0.001, and 49% vs. 69%; *P* < 0.001, respectively). Moreover, nodal status was lower (N0 55% vs. 25%, N1 38% vs. 39%, and N2 8% vs. 36%; *P* < 0.001), and a higher rate of R0 resections was observed (64% vs. 45%; *P* < 0.001). Lastly, adjuvant chemotherapy was more often administered (75% vs. 64%; *P* = 0.03) (Table 1).

### Prognostic Factors

Because of nonproportionality, adjuvant chemotherapy was included as a time-varying covariate. Factors independently associated with long-term DFS were low preoperative serum CA 19-9 level (logarithmic; HR 1.21; 95% CI 1.10–1.32; *P* < 0.001), absence of vascular resection HR 1.33; 95% CI 1.12–1.58; *P* < 0.001), T1 tumor stage (vs. T3; HR 1.52; 95% CI 1.14–2.04; *P* = 0.005), well/moderate tumor differentiation (vs. poor; HR 1.44; 95% CI 1.22–1.68; *P* < 0.001), absence of perineural invasion (HR 1.42; 95% CI 1.11–1.81; *P* = 0.006), N0 and N1 lymph node status (vs. N2; HR 1.92; 95% CI 1.54–2.40; *P* < 0.001, and HR 1.33; 95% CI 1.11–1.60; *P* = 0.002, respectively), R0 resection margin status (vs. R1; HR 1.25; 95% CI 1.07–1.46; *P* = 0.005), and adjuvant chemotherapy (HR 1.74; 95% CI 1.47–2.06; *P* < 0.001). Sex (male vs. female), CACI (< 4 vs. ≥ 4), neoadjuvant therapy (yes vs. no), tumor stage (T2 vs. T3), lymphovascular invasion (no vs. yes), and major complications (no vs. yes) were not independently associated with long-term DFS (Table 2). Complementary to whether a patient started adjuvant chemotherapy, the number of adjuvant chemotherapy cycles was also independently associated with long-term DFS (HR 1.12; 95% CI 1.09–1.15; *P*<0.001).

**TABLE 2:** Multivariable Cox Proportional Hazard Analysis to Identify Independent Predictors of Long-term Disease-free Survival of ≥5 Years After Resection of Pancreatic Ductal Adenocarcinoma

	HR (95% CI)	*P*
Sex (male vs. female)	1.01 (0.87–1.18)	0.88
CACI (<4 vs. ≥4)	0.99 (0.85–1.15)	0.90
Neoadjuvant therapy (yes vs. no)	1.05 (0.79–1.40)	0.73
Preoperative CA 19-9 level (logarithmic)^*^	1.21 (1.10–1.32)	<0.001
Vascular resection (no vs. yes)	1.33 (1.12–1.58)	<0.001
Tumor stage 8th AJCC edition		
T1	1.52 (1.14–2.04)	0.005
T2	1.17 (0.98–1.39)	0.08
T3	Ref (Ref)	Ref
Tumor differentiation (well/moderate vs. poor)	1.44 (1.22–1.68)	<0.001
Perineural invasion (no vs. yes)	1.42 (1.11–1.81)	0.006
Lymphovascular invasion (no vs. yes)	1.14 (0.96–1.36)	0.14
Lymph node status 8th AJCC edition		
N0	1.92 (1.54–2.40)	< 0.001
N1	1.33 (1.11–1.60)	0.002
N2	Ref (Ref)	Ref
Resection margin status (R0≥1 mm vs. R1<1 mm)	1.25 (1.07–1.46)	0.005
Major complications (no vs. yes)	1.14 (0.97–1.35)	0.12
Adjuvant chemotherapy (yes vs. no)	1.74 (1.47–2.06)	< 0.001

* The corresponding hazard ratio indicates the risk if the preoperative CA 19-9 level decreases by 1 log-unit.

AJCC indicates American Joint Committee on Cancer; CA 19-9, carbohydrate antigen 19-9; CACI, Charlson Age-adjusted Comorbidity Index; 95% CI, 95% confidence interval; HR, hazard ratio; P, probability-value.

### Predictive Model

**FIGURE 1:**
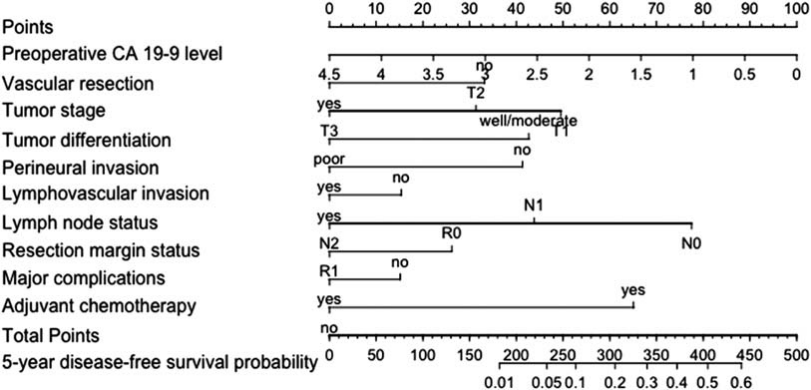
Nomogram to predict the probability of disease-free survival of ≥ 5 years for individual patients after resection of pancreatic ductal adenocarcinoma.

Based on AIC, 10 out of 13 variables were selected for the final predictive model: preoperative CA 19-9 level, vascular resection, tumor stage, tumor differentiation, perineural invasion, lymphovascular invasion, lymph node status, resection margin status, major complications, and adjuvant chemotherapy (Fig. 1). The *C*-index of the final model was 0.68, which did not change after internal validation. The calibration slope was 0.99 (Supplemental Fig. 1, Supplemental Digital Content 1, http://links.lww.com/SLA/E729). Each variable was scored between 0 and 100 points, which led to a total score between 0 and 466 points (Supplemental Table 2, Supplemental Digital Content 1, http://links.lww.com/SLA/E729).

### Sensitivity Analysis

Of the 118 long-term survivors, 36 patients (31%) were confirmed long-term survivors based on imaging or CA 19-9, while 82 patients (69%) did not experience symptoms of disease recurrence and were therefore considered disease free. The final predictive model had a *C*-index of 0.67 and a calibration slope of 0.98 when evaluated among the confirmed long-term disease-free survivors.

## DISCUSSION

In this study, the first model to predict long-term DFS was designed and internally validated using data from a nationwide, unselected cohort of patients. This model, including preoperative serum CA 19-9, vascular resection status, tumor stage, tumor differentiation, perineural invasion, lymphovascular invasion, nodal stage, resection margin status, major complications, and adjuvant chemotherapy, may aid in prognostication and shared decision-making for individual patients after resection of PDAC.

Regarding the identification of prognostic factors, multiple studies have been performed to identify factors associated with disease recurrence in general. For example, increased preoperative serum CA 19-9, poor differentiation grade, vascular resection, lymphovascular invasion, R1 resection margin status, higher nodal status and ratio, high tumor size/stage, and omission of adjuvant chemotherapy have been suggested to negatively impact DFS.^8,12^ Studies that reported on the cut-off at 5-year DFS after resection of PDAC specifically mainly focused on identifying factors associated with an increased risk of developing disease recurrence within these first 5 years. One monocenter study including 768 patients found higher age, larger tumor size, poor tumor differentiation, and lymph node metastases to be independently associated with an increased risk of developing disease recurrence within 5 years after resection. In contrast to the results of the current study, higher preoperative serum CA 19-9, vascular resection, poor resection margin status, and omission of adjuvant chemotherapy were not independently associated with disease recurrence within 5 years postoperative.^10^ Only 1 study investigated factors associated with long-term DFS, which solely identified the absence of perineural invasion as an independent predictor. Sex, preoperative serum CA 19-9, vascular resection, tumor size, nodal status, tumor differentiation, lymphovascular invasion, and resection margin status were also evaluated but appeared to be irrelated to 5-year DFS.^11^ The fact that the association of these factors was not significant, which conflicts with some of the results of our study, could be attributed to the limited sample size of only 176 patients. Specifically, there was a small number of long-term disease-free survivors (ie, 20 patients (11%) vs. 118 (14%) in our study). As disease recurrence is the most frequent cause of death in patients who underwent resection of PDAC, DFS may be considered a precursor of OS. Therefore, the association between prognostic factors and long-term OS is also of interest. A study investigating this association only identified nodal status as an independent prognostic factor, although tumor size, number of positive lymph nodes, and resection margin status were also included in the multivariable analysis.^24^ In the current study, a more extensive set of predictive factors was found, which were subsequently combined in a prognostic model which can be easily used in clinical practice.

The proportion of long-term survivors in this study was in line with the survival rates reported in previous literature.^10,11^ However, studies that investigated long-term DFS included patients who underwent resection several years ago since sufficient follow-up of considerable years is essential. Over the past years, advancements have been made in pancreatic cancer treatment. Adjuvant treatment strategies have developed, with a shift from primarily adjuvant gemcitabine monotherapy to more effective gemcitabine combination regimens or adjuvant FOLFIRINOX chemotherapy.^4,25^ In addition, results from clinical trials suggested improved survival in patients who were treated with neoadjuvant strategies, which are increasingly applied in current clinical practice.^26–28^ It is reasonable to believe these changes in (neo)adjuvant treatment will also affect 5-year DFS rates, but this remains to be evaluated in future studies.

Even though the factors identified in this study are predictive of long-term DFS, long-term survivors in our cohort did also occasionally have unfavorable clinical prognostic factors. This suggests that besides these identified clinical factors, there are unidentified factors regarding, for instance, tumor biology and genetics that influence the probability of long-term DFS. Although this has been the subject of former research, these factors remain largely unknown.^29^ Molecular aspects predicting disease recurrence have been the subject of previous studies.^30^ Multiple genetic alterations were found to be related to PDAC, although mutation analysis alone cannot adequately predict long-term DFS.^29,31^ RNA transcriptional analysis has revealed molecular subgroups of PDAC, suggesting that other factors regulating gene expression, such as epigenetics, explain intertumoral heterogeneity.^32^ Therefore, epigenetic analysis through whole genome DNA methylation profiling may reveal molecular subsets associated with disease recurrence and long-term survival. Also, altered gene expression results in altered protein expression, and most diseases are manifested at the level of protein activity. Therefore, proteomic research could provide a better comprehension of the precise cascade leading to disease recurrence and potentially clarify their impact on the timing of PDAC recurrence.

Although the exact underlying biology remains largely unknown, patients who develop disease recurrence at a late point in time after resection of PDAC are assumed to have less aggressive tumor biology. A previous study showed that the DFS of patients who develop isolated local or isolated lung recurrence is significantly longer than the DFS of patients who develop recurrence in the liver. In addition, long-term survivors who still develop disease recurrence recur locally in two-thirds of cases.^8^ This suggests that the timing and location of disease recurrence are important factors influencing patients’ survival. Patients with favorable recurrence locations, such as local-only and lung-only, might specifically benefit from recurrence-focused treatment aiming to prolong survival. Consequently, it might be relevant to identify these patients with a high probability of long-term DFS that might specifically benefit from intensified postoperative monitoring with the goal to diagnose and treat recurrence as soon as possible. On the contrary, one could argue that patients with a high probability of long-term DFS can receive less frequent follow-up since their tumor biology is likely to be less aggressive. Nevertheless, the true value of standardized surveillance after resection of PDAC has yet to be determined. To this end, the Dutch Pancreatic Cancer Group is currently performing the RADAR-PANC trial on the additional value of a 3-monthly standardized surveillance with imaging and tumor marker testing (NCT04875325).

This study evaluated a large, multicenter, nationwide cohort with sufficient follow-up to investigate long-term DFS. However, some limitations should be acknowledged when interpreting these results. First, although baseline and perioperative data were collected prospectively, data on follow-up and recurrence were retrospectively collected from the patients’ medical records. Second, a standardized follow-up strategy could have influenced the timing of the recurrence diagnosis. However, standardized surveillance was solely conducted in the context of participation in prospective trials with long-term oncological endpoints, which was only the case in ~10% of patients in this study. Also, study-specific follow-up is often limited to the maximum duration of around 2 years, which therefore could not have influenced if patients were determined long-term disease-free survivors or not. Third, patients who are in worse physical condition after their resection, for instance, due to postoperative complications, might more often be unwilling or unable to receive adjuvant chemotherapy. As a result, these patients may be discharged from follow-up, causing differential losses to follow-up. Nevertheless, the small number of missing data in this study, of which none in the primary outcome, is unlikely to have influenced the results. Fourth, the developed predictive model had a *C*-index of 0.68, which corresponds to moderate discriminative ability. Since the model will have no stand-alone consequences, we believe this will suffice for the prognostication of patients. Fifth, only a small subset of patients received neoadjuvant treatment. Therefore, the association between neoadjuvant therapy and long-term DFS could be underestimated because of a power issue. Also, the model was developed to be easily applicable to clinical practice. Therefore, non-secretors of CA 19-9 remained included in the analysis, and their prognostication can be slightly overestimated because of the points rewarded for a low CA 19-9 value. Lastly, although the model was internally validated, additional external validation is necessary to determine its true prognostic value.

In conclusion, this nationwide, multicenter observational study identified factors that are associated with long-term DFS after resection of PDAC. These variables were combined in a prediction model made available at www.pancreascalculator.com, which may aid in prognostication and shared decision-making for individual patients.

## Supplementary Material
