# Supplementary material for: Predicting Long-term Disease-free Survival After Resection of Pancreatic Ductal Adenocarcinoma: A Nationwide Cohort Study
Source: Ann Surg. 2023 Jul 17;279(1):132–7. doi: 10.1097/SLA.0000000000006004 (PMC10727199; doi:10.1097/SLA.0000000000006004)
Supplement: Supplementary file 1 [file sla-279-132-s001.docx]

| ***Supplemental table 1****. Baseline characteristics and missing data of 836 patients who underwent resection of pancreatic ductal adenocarcinoma* | | |
| --- | --- | --- |
|  | | **Missing, n (%)** |
| Age at diagnosis, mean (SD), in years | 67 (9) | 0 (0) |
| Male sex, n (%) | 459 (55) | 0 (0) |
| BMI, mean (SD), kg/m^2^ | 25 (4) | 2 (0) |
| CACI, n (%) |  | 0 (0) |
| < 4 | 428 (51) |  |
| ≥ 4 | 408 (49) |  |
| ASA-score, n (%) |  | 12 (1) |
| I | 122 (15) |  |
| II | 520 (62) |  |
| III | 181 (22) |  |
| IV | 1 (0) |  |
| ECOG performance score at primary diagnosis, n (%) |  | 307 (37) |
| 0 | 230 (28) |  |
| 1 | 239 (29) |  |
| 2 | 46 (6) |  |
| 3 | 13 (2) |  |
| 4 | 1 (0) |  |
| Neoadjuvant therapy, n (%) |  | 1 (0) |
| None | 763 (91) |  |
| FOLFIRINOX chemotherapy | 32 (4) |  |
| Gemcitabine chemoradiotherapy | 40 (5) |  |
| Preoperative serum CA 19-9 level, median (IQR), U/mL | 127 (33-485) | 266 (32) |
| Type of surgery, n (%) |  | 2 (0) |
| Open | 753 (90) |  |
| Laparoscopic | 75 (9) |  |
| Robot-assisted | 6 (1) |  |
| Surgical procedure, n (%) |  | 0 (0) |
| Pancreatoduodenectomy | 683 (82) |  |
| Distal pancreatectomy | 116 (14) |  |
| Total pancreatectomy | 37 (4) |  |
| Tumor location, n (%) |  | 0 (0) |
| Head | 712 (85) |  |
| Body/tail | 124 (15) |  |
| Vascular resection, n (%) | 225 (27) | 2 (0) |
| Tumor stage 8^th^ AJCC edition, n (%) |  | 17 (2) |
| T1 | 91 (11) |  |
| T2 | 515 (62) |  |
| T3 | 213 (25) |  |
| Tumor differentiation, n (%) |  | 92 (11) |
| Well/moderate | 511 (61) |  |
| Poor | 233 (28) |  |
| Microscopic lymphovascular invasion, n (%) | 429 (51) | 193 (23) |
| Microscopic perineural invasion, n (%) | 652 (78) | 98 (12) |
| Lymph node stage 8^th^ AJCC edition, n (%) |  | 3 (0) |
| N0 | 243 (29) |  |
| N1 | 326 (39) |  |
| N2 | 264 (32) |  |
| Total resected lymph nodes, median (IQR) | 15 (10-20) | 12 (1) |
| Lymph node ratio, n (%) |  |  |
| ≤ 0.2 | 514 (61) |  |
| > 0.2 | 310 (37) |  |
| TNM stage 8^th^ AJCC edition, n (%) |  | 11 (1) |
| ≤ 2A | 238 (28) |  |
| ≥ 2B | 587 (70) |  |
| Major postoperative complications^a^, n (%) | 250 (30) | 0 (0) |
| Hospital stay, median (IQR), days | 11 (8-16) | 0 (0) |
| Resection margin status, n (%) |  | 9 (1) |
| R0 > 1 mm | 397 (47) |  |
| R1 ≤ 1 mm | 430 (51) |  |
| Adjuvant chemotherapy, n (%) | 531 (64) | 27 (3) |
| Type adjuvant chemotherapy, n (%) |  | 7 (1) |
| Gemcitabine monotherapy | 495 (93) |  |
| FOLFIRINOX | 15 (3) |  |
| Gemcitabine combination therapy | 12 (2) |  |
| Other | 2 (1) |  |
| Use of CA 19-9 during follow-up, n (%) |  | 91 (11) |
| No | 239 (29) |  |
| Yes, not routinely | 422 (50) |  |
| Yes, routinely | 84 (10) |  |
| Use of imaging procedures during follow-up, n (%) |  | 72 (9) |
| No | 60 (7) |  |
| Yes, not routinely | 594 (71) |  |
| Yes, routinely | 110 (13) |  |
| Percentages may not add up to 100 because of rounding.  ^a^ Major postoperative complications were defined as complications requiring surgical or radiologic intervention, intensive care unit admittance, single- or multi-organ failure, of the patients’ demise.  AJCC: American Joint Committee on Cancer; ASA: American Society of Anesthesiologists; BMI: body mass index; CA 19-9: Carbohydrate Antigen 19-9; CACI: Charlson Age-adjusted Comorbidity Index; CI: confidence interval; ECOG: Eastern Cooperative Oncology Group; FOLFIRINOX: fluorouracil, leucovorin, irinotecan, oxaliplatin; IQR: Interquartile range; SD: standard deviation. | | |

| ***Supplemental table 2****. Risk table of the final prediction model for long-term disease-free survival of ≥ five years after resection of pancreatic ductal adenocarcinoma* | |
| --- | --- |
|  | **Risk score** |
| Preoperative CA 19-9 level (logarithmic) |  |
| 0 | 100 |
| 0.5 | 89 |
| 1 | 78 |
| 1.5 | 67 |
| 2 | 56 |
| 2.5 | 44 |
| 3 | 33 |
| 3.5 | 22 |
| 4 | 11 |
| 4.5 | 0 |
| Vascular resection |  |
| No | 33 |
| Yes | 0 |
| Tumor stage 8^th^ AJCC edition |  |
| T1 | 50 |
| T2 | 31 |
| T3 | 0 |
| Tumor differentiation |  |
| Well/moderate | 43 |
| Poor | 0 |
| Perineural invasion |  |
| No | 41 |
| Yes | 0 |
| Lymphovascular invasion |  |
| No | 15 |
| Yes | 0 |
| Lymph node status 8^th^ AJCC edition |  |
| N0 | 78 |
| N1 | 44 |
| N2 | 0 |
| Resection margin status |  |
| R0 ≥ 1 mm | 26 |
| R1 < 1 mm | 0 |
| Major complications |  |
| No | 15 |
| Yes | 0 |
| Adjuvant chemotherapy |  |
| No | 0 |
| Yes | 65 |
| AJCC: American Joint Committee on Cancer; CA 19-9: Carbohydrate Antigen 19-9. | |


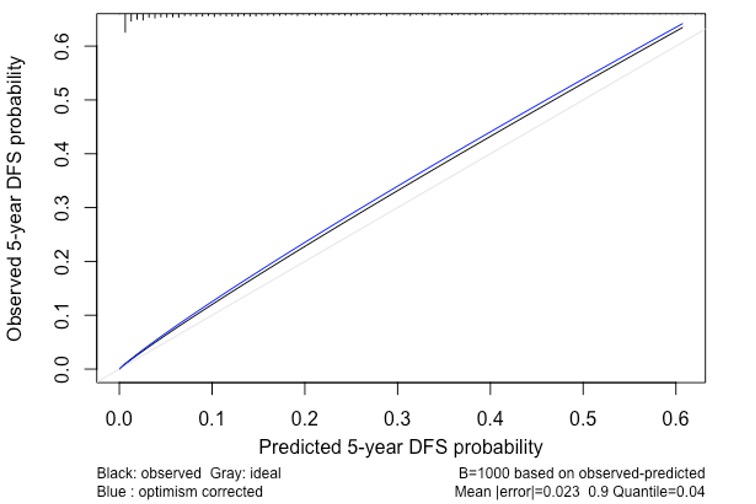


Supplementary Figure 1: Calibration plot of final predictive model
